# Cell‐cycle‐specific lesion evolution rather than inhibition of double‐strand‐break repair underpins cisplatin radiosensitization

**DOI:** 10.1002/1878-0261.70240

**Published:** 2026-03-19

**Authors:** Ye Qiu, Xixi Lin, Emil Mladenov, Veronika Mladenova, Jürgen Thomale, Ali Sak, Yao Wang, Yunxuan Deng, Eleni Gkika, Martin Stuschke, George Iliakis

**Affiliations:** ^1^ Division of Experimental Radiation Biology, Department of Radiation Therapy University Hospital Essen, University of Duisburg‐Essen Germany; ^2^ Institute of Medical Radiation Biology University Hospital Essen, University of Duisburg‐Essen Germany; ^3^ Institute of Cell Biology University Hospital Essen Germany; ^4^ Radiation Biology Laboratory, Department of Radiotherapy and Radiation Oncology University Hospital Bonn Germany; ^5^ German Cancer Consortium (DKTK) Partner Site University Hospital Essen, German Cancer Research Center (DKFZ) Germany

**Keywords:** cell cycle, cisplatin, cisplatin‐DNA adducts, DNA double‐strand breaks, DNA repair, radiosensitization

## Abstract

DNA double‐strand breaks (DSBs) generated from collisions of DNA replication forks with cisplatin‐induced interstrand crosslinks underpin cisplatin cytotoxicity. Yet, the impact of cell‐cycle‐dependent cisplatin–DNA adduct (CDA) formation on cell cycle progression and interactions with DNA replication remain incompletely characterized. Also, although cisplatin enhances tumor response to ionizing radiation (IR), the underpinning radiosensitizing mechanisms remain unresolved. Here, we close this void and analyze CDAs (GpG crosslinks) and DSB (γH2AX foci) induction and repair in a strictly cell‐cycle‐dependent manner. We report for the first time that CDAs form in a dose‐ and time‐dependent manner in all cell cycle phases, whereas DSBs emerge only in S‐phase. Repair of IR‐induced DSBs remains largely unaffected by CDAs in G_1_, S, and G_2_ phases, but is inhibited when S‐phase‐treated cells progressed to G_2_. Radiosensitization occurs after prolonged cisplatin exposure, likely owing to G_2_‐phase accumulation and lesion evolution from S‐phase, thus impairing repair of IR‐induced DSBs. Cisplatin fails to radiosensitize quiescent (G_0_) cells. In summary, CDA formation is similar across the cell cycle, but DSBs form only in S‐phase. Whereas CDAs fail to interfere with repair of IR‐induced DSBs, progression‐dependent repair disruptions cause radiosensitization. Elucidation of the underpinning mechanisms may help to design improved cisplatin–radiation schedules for more efficacious therapies.

## Graphical abstract

We analyze cisplatin–DNA adducts (CDAs) and double‐strand breaks (DSBs) in a cell‐cycle‐dependent manner. We find that CDAs form similarly across all cell cycle phases. DSBs arise only in S‐phase. CDAs might not directly impair DSB repair, but S‐phase DSB lesions evolve in the presence of CDAs and disrupt repair in G2, also causing radiosensitization.

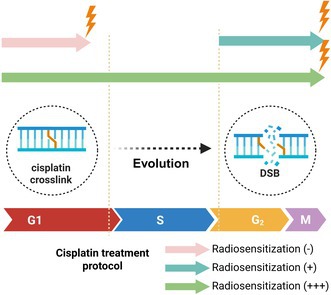

**Abbreviations**
CDA: (cisplatin–DNA adduct)
DSBs: (DNA double‐strand breaks)
EdU: (5‐ethynyl‐2‐deoxyuridine)
HR: (homologous recombination)
ICL: (interstrand crosslinks)
IR: (ionizing radiation)
NHEJ: (non‐homologous end‐joining)
QIBC: (quantitative image‐based cytometry)
RT: (radiotherapy)

## Introduction

Cisplatin (cis‐diamminedichloroplatinum II) is the cornerstone of several cancer treatment protocols. Indeed, cisplatin‐based concurrent chemoradiotherapy is recommended in treatment guidelines for various cancers [1, 2, 3]. Cisplatin is also the most commonly used agent for enhancing the therapeutic effects of radiotherapy (RT). Studies on locoregionally advanced nasopharyngeal carcinoma have demonstrated that the 5‐year overall survival rate with cisplatin‐based concurrent chemoradiotherapy can reach 60%–72%, a significant improvement over RT alone (~54%); the treatment also reduces the risk of local recurrence and distant metastasis [2, 4, 5]. In patients with nonmetastatic inoperable non‐small cell lung cancer (NSCLC), the concomitant use of cisplatin has increased the 3‐year overall survival by 14% compared to RT alone [3].

The analysis of possible synergisms between cisplatin and RT has also been the focus of extensive research on the underpinning mechanisms, but as of yet no definitive answers have emerged. One frequently envisioned mechanism is that the combination of cisplatin with ionizing radiation (IR) enhances the cytotoxic consequences of DNA damage, particularly DSBs, induced by either monotherapy. However, whether such synergisms indeed occur and the mechanistic basis that underpins them remain speculative.

It is widely assumed that DNA is the critical target for the cell killing induced by both cisplatin and IR. Upon uptake via ion transporters, cisplatin undergoes aquation and binds to the N^7^ position of guanine on DNA, forming intrastrand 1,2‐d(GpG), ‐d(ApG), 1,3‐d(GpXpG) adducts and interstrand G‐G cross‐links (ICLs). These DNA‐cisplatin adducts (CDAs) distort the DNA helical structure and disrupt DNA replication and transcription [6, 7, 8]. IR produces a variety of DNA lesions including base damage, apurinic/apyrimidinic (AP) sites, DNA single‐strand breaks (SSBs), and double‐strand breaks (DSBs). Among these lesions, DSBs are widely considered the most deleterious, and the outcome of their repair is a critical determinant of cell death [9].

In the field of classic radiosensitizers, radiosensitization is attributed to inhibition of one or more DSB repair pathways. Homologous recombination (HR), non‐homologous end‐joining (NHEJ), alternative end‐joining (alt‐EJ), and single‐strand annealing (SSA) are the pathways utilized by cells to process DSBs (reviewed in [9, 10]). Early studies have suggested that cisplatin can inhibit NHEJ when CDAs form in close proximity to IR‐induced DSBs [11, 12, 13]. They can impede the translocation of Ku along the DNA strand and thus reduce DNA‐PK kinase activity, a crucial step for initiating NHEJ [14, 15, 16, 17]. In addition, the presence of CDAs has been shown to obstruct the movement of exonucleases along the DNA strand [12], suggesting that cisplatin also disrupts the nucleolytic processing of DNA ends to generate 3′‐ssDNA overhangs (DNA end resection). Such impairment of resection would suppress all resection‐dependent DSB repair pathways: HR, SSA, and alt‐EJ.

Notably, the above proposed mechanisms consider processing of DSBs with proximal CDAs. However, such coincidence is expected to be very rare at the cisplatin concentrations and IR doses typically used experimentally and in the clinic (< 0.2% frequency) [12], and is therefore unlikely to be the main mechanism of cisplatin‐radiation interactions. Other experiments show that cisplatin by itself fails to impair end‐joining of non‐cisplatin‐damaged DNA [13], and that it also fails to affect global DSB repair kinetics in the cellular context [18, 19]. Thus, the actual mechanism of cisplatin radiosensitization remains unknown.

Compared to cisplatin‐induced intrastrand crosslinks, ICLs, although accounting for less than 5% of total cisplatinated DNA [20, 21], are significantly more cytotoxic especially for proliferating cells. This heightened cytotoxicity arises because ICLs bind covalently the two strands of the DNA helix to each other and prevent all scheduled metabolic processes that require DNA strand separation. Indeed, replication of DNA containing ICLs leads to DNA replication fork collapse and the formation of DSBs [22, 23, 24]. Therefore, cisplatin cytotoxicity correlates with the amount of ICLs present in S phase [24, 25] and is highest in cells that are about to enter S phase than in cells that are about to exit S phase [26]. Furthermore, cell cycle progression determines the degree of cisplatin‐induced apoptosis, and exposure during the G_2_/M phase results in a higher apoptotic index and earlier PARP cleavage, a marker of apoptosis [27], as compared to treatment during the G_1_ phase [27]. This sequence of events can be accelerated by the abrogation of cell cycle checkpoints, further enhancing cisplatin's efficacy [28, 29, 30, 31].

It has long been theorized that cisplatin exerts synergistic effects with radiation by preferentially killing cells in the S phase and inducing cell cycle arrest at the G_2_/M phase. This is because cells are most resistant to radiation during the late S phase and most sensitive during mitosis and the late G_2_ phase [32, 33, 34]. The above properties of cisplatin imply that its interaction with IR is likely to be indirect and cell‐cycle‐dependent, deriving from effects in only a fraction of cells.

Two forms of cisplatin‐radiation interactions can be postulated: (i) The presence of CDAs affects how cells repair IR‐induced DSBs in the different phases of the cell cycle. (ii) The presence of IR‐induced DSBs affects the cytotoxic consequences of CDAs. In the present report, we focus on radiation response and delve into the first form of possible interactions: how the progression of CDA‐containing cells through the cell cycle influences the processing of IR‐induced DSBs. To comprehensively address this scientific question, we begin with a detailed analysis of CDA formation patterns using an antibody raised against cisplatin adducts and examine levels of adduct formation throughout the cell cycle with a high‐throughput, cell‐cycle‐specific analysis. We further examine DSB formation and repair after exposure to cisplatin in combination with radiation. Finally, we analyze cisplatin‐induced radiosensitization in cells enriched in specific phases of the cell cycle.

We show that cisplatin induces DSBs specifically in S phase, and that the presence of CDAs in any phase of the cell cycle has a negligible effect on the repair of IR‐induced DSBs. Finally, we highlight a crucial role for cell cycle progression in determining cisplatin‐mediated effects on DSB repair and radiosensitization.

## Materials and methods

### Cell lines and growth conditions

Two human tumor cell lines were selected for the experiments of the present work to confirm the validity of key findings. The human A549 (RRID:CVCL_0023) and NCI‐H460 (RRID:CVCL_0459) non‐small cell lung carcinoma (NSCLC) cell lines were obtained from ATCC (Rockville, MD, USA). A549 and H460 cells were cultured in McCoy's 5A medium with 10% FBS and antibiotics. Both cell lines were maintained at 37 °C in 95% air and 5% CO_2_. Cells were typically re‐thawed every 3 months to reduce the probability of genetic shifts owing to inherent genomic instability. Cell lines were authenticated using Multiplex Cell Authentication by Multiplexion and confirmed mycoplasma‐free before freezing using the MycoAlert Plus Mycoplasma detection kit from Lonza (LT07‐705).

### Drugs and antibodies

Details are provided in Table S1.

### Radiation exposure

Radiation was delivered using an Isovolt‐320 kV X‐ray tube (Precision) at a 500 mm distance, with a 1.65 mm aluminum filter, a dose rate of ~3.25 Gy·min^−1^ and an effective photon energy of ~90 keV.

### Clonogenic survival assay

Cells were plated at 2 × 10^5^ per 60 mm dish with 5 mL of growth medium and incubated for 48 h to achieve exponential growth. Radiation, when prescribed by the protocol, was administered after varying durations of cisplatin treatment (details in the figure legends). Cells were plated in triplicate, transferred to culture boxes with water to maintain moisture and reduce environmental toxicity, and incubated at 37 °C in a 95% air/5% CO_2_ atmosphere for 10–14 days. Colonies were stained with 0.5% crystal violet, with clusters of at least ~50 cells scored as colonies. The cell survival was calculated as the ratio between colonies counted and cells plated, corrected by the plating efficiency.

### Cell cycle analysis using flow cytometry (FC) and immunofluorescence (IF)

Cells were pulse‐labeled with EdU (5‐ethynyl‐2‐deoxyuridine) for 30 min to label S‐phase cells, followed by treatments as required by the experiment. After collection, cells were permeabilized with ice‐cold PBS containing 0.25% Triton™ X‐100 for 5 min, fixed with 3% paraformaldehyde and 2% sucrose in PBS for 15 min, and blocked overnight at 4 °C with PBG blocking buffer (0.2% Gelatin, 0.5% BSA in PBS). EdU signal development was performed using an EdU Click‐IT staining kit (ThermoFisher Scientific) as per the manufacturer's protocol. Cells were then stained with 1 μg·mL^−1^ propidium iodide (PI) in PBS for 15 min at 37 °C. Cell cycle distributions were obtained using a Gallios flow cytometer (Beckman Coulter) and analyzed with Kaluza software (Beckman Coulter).

The formation and decay of γH2AX and 53BP1 foci were measured by IF coupled with QIBC analysis, while γH2AX and Ki‐67 signal intensity were detected by FC. The experimental design and analysis were described previously [35, 36].

### Quantification of CDAs

CDAs in NSCLC cells were quantified using an immuno‐cytological assay (ICA) with optimizations as described previously [19, 37, 38, 39, 40]. Cells were pulse‐labeled with 2 μm EdU for 30 min, then treated with varying cisplatin concentrations for 1 h. After collection, cell pellets were diluted in cold PBS, dropped onto Superfrost Gold Slides (Thermo Fisher) and air‐dried. The slides were fixed in ice‐cold methanol for 15 min at −20 °C, followed by alkaline denaturation (70 mm NaOH, 140 mm NaCl in 40% methanol, 0 °C, 5 min) and proteolytic digestion with 60 μg·mL^−1^ pepsin (10 min at 37 °C), and 40 μg·mL^−1^ proteinase K (10 min at 37 °C). Cells were then blocked in PBG buffer (0.2% fish skin gelatin, 0.5% BSA in PBS) for 1–2 h at room temperature, followed by incubation with a rat primary antibody (1 : 500) recognizing cisplatin‐GpG DNA adducts (R‐C18) overnight at 4 °C. Subsequently, anti‐rabbit IgG Alexa Fluor 488 secondary antibody (1 : 400) was applied for 1 h. DAPI nuclear counterstaining (1 μg·mL^−1^) was performed for 1 h at 37 °C. Images were acquired using an AxioScan.Z1 high‐throughput fluorescence microscope (QIBC) (Carl Zeiss GmbH, Göttingen, Germany) and were analyzed in a cell‐cycle‐specific manner using Imaris and Orange software.

### Synchronization in the cell cycle

A549 cells were synchronized at G_1_/S border using a single thymidine block. 0.2 million cells were plated per 60 mm dish/75 mm flask and cultured for 24 h before thymidine treatment (2 mm, 20 h). Following two washes with pre‐warmed PBS, cells were released in complete fresh medium. S‐phase enrichment occurred at 2 h post‐release and G_2_‐phase at 6 h post‐release. To delay the progression of cells through the cell cycle, enriched cells were incubated in a balanced salt solution (BSS) [41] containing per liter: 6.41 g NaCl, 0.4 g KCl, 0.2 g MgSO_4_·7H_2_O, 0.58 g NaH_2_PO_4_·H_2_O, 2.2 g NaHCO_3_ and 0.14 g CaCl_2_. This BSS has a salt composition similar to that of the McCoy's 5A medium and was found to give optimal cell viability.

Serum deprivation was used to enrich cells in G_1_/G_0_. 0.1 million A549 cells were plated per 60 mm dish, grown for 2 days, then switched to serum‐free medium for 3 more days, as described elsewhere [42], before use in experiments. Details on radiation and cisplatin exposure on such, cell cycle phase enriched cells, are given in the corresponding figure legends.

### Mitotic index analysis using FC

Two parametric FC analyses were employed to simultaneously measure DNA content by PI and cells in mitosis by detection of phosphorylated Histone H3 at Serine 10 (H3‐pS10). The experimental design and analysis were described previously [43]. Proper gating was applied to select H3‐pS10 positive events that represented cells in mitosis. The mitotic index (MI) was calculated as the fraction of cells in mitosis.

### Pulsed‐field gel electrophoresis (PFGE)

PFGE was used to assess the induction and repair of DSBs as described elsewhere [43, 44]. In brief, the DSB load in cells is measured by the fraction of DNA released (FDR) out of the well into the lane of an agarose gel. Dose response curves are plotted as FDR versus IR dose. Fitted lines are used to calculate the equivalent Gy‐dose values (DEQ) for each FDR measured at a given repair time point. Repair kinetics are shown as plots of DEQ versus time.

### Statistical analysis

We assessed the normality of differences between groups using the Shapiro–Wilk test. For normally distributed differences (*P* > 0.05), we used a paired two‐tailed Student's *t*‐test to compare means between the two time points. For non‐normally distributed data, the non‐parametric Wilcoxon signed‐rank test was applied. Data are presented as mean ± SEM of biological replicates. All analyses were performed using GraphPad Prism software (version 9.0). Statistical significance is indicated as *P < 0.05, **P < 0.01, or ***P < 0.001.

## Results

### Cisplatin ‐DNA adducts form at similar yields in different phases of the cell cycle

**Fig. 1:**
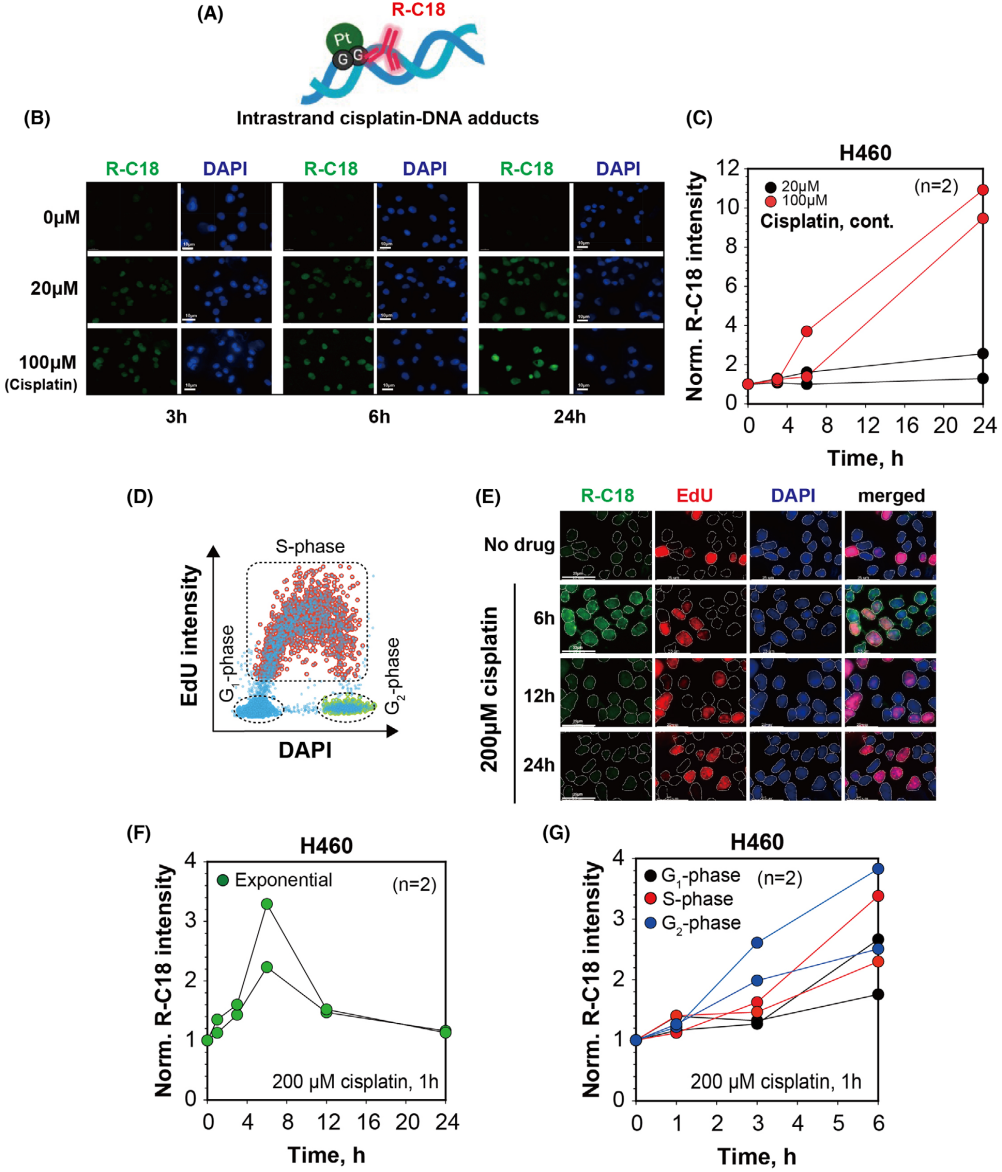
The formation of cisplatin‐DNA adducts throughout the cell cycle. (A) Binding specificity of the R‐C18 antibody detecting Pt‐[GG] (guanine‐guanine intrastrand crosslinks); (B) Representative images of GpG adducts in H460 cells. Scale bar: 10 μm; (C) Quantification of R‐C18 signal intensity in exponentially growing H460 cells continuously treated with varying concentrations of cisplatin; (D) Representative histogram and dot plots obtained from QIBC (quantitative image‐based cytometry); histogram shown is representative of two independent experiments with similar results; (E) Representative images of GpG adducts co‐localized with EdU in H460 cells. Scale bar: 25 μm; (F) Induction and resolution kinetics of GpG adducts in H460 cells following a 1 h exposure to 200 μm cisplatin; (G) Cell cycle‐specific quantification of R‐C18 signal intensity in cells treated as in (F). Intensity data were normalized to the value measured in untreated controls. Each data point represents an independent experiment.

Because cisplatin toxicity is confined to S‐phase of the cell cycle [6, 38], we inquired whether this specificity partly reflects enhanced CDA formation during S‐phase. This facet of cisplatin action has not been systematically investigated previously, although it is relevant for the mechanistic understanding of its cytotoxicity. To address this question, we quantified CDAs in individual cells using a monoclonal antibody, R‐C18, specifically raised against guanine‐guanine (Pt‐[GG]) intrastrand crosslinks [38] (Fig. 1A). Although intrastrand crosslinks are much less cytotoxic than ICLs, they are ~20× more abundant, which facilitates detection. Moreover, they form at fixed ratios to the highly cytotoxic ICLs [38], and can be used as a proxy for ICLs.

For cell‐cycle‐specific analysis of CDAs, we combined IF detection with DNA content analysis by means of DAPI staining. To improve the statistical power of our analyses, we adopted a high‐throughput quantitative image‐based analysis platform (QIBC) that allowed simultaneous quantification of R‐C18 and DAPI signals at each time point and drug dose in several thousands of cells. The combination of this unique detection methodology with high‐throughput analysis allowed us to quantitatively investigate for the first‐time key questions of cisplatin treatment and to obtain valuable mechanistic insights into its cytotoxic effects.

To standardize detection of CDAs with the R‐C18 antibody, we exposed H460 cells, grown on coverslips, continuously to 0, 20, or 100 μm cisplatin and stained for R‐C18 signal at 3, 6, and 24 h later. Although cells were counterstained with DAPI, this signal was not considered in this experiment, and all cells were pooled and analyzed for CDA formation. Figure 1B shows a clear increase in R‐C18 signal intensity with increasing treatment time and drug concentration. It is evident that the R‐C18 signal was detected in all cells. CDA formation was a relatively slow process, with only a small amount of adducts forming at 3–6 h, but with many more present at 24 h (Fig. 1C).

We continued with cell cycle‐specific adduct detection in single cells by integrating in the QIBC analysis of DAPI, EdU and R‐C18 signals, using the gates for DAPI and EdU shown in Fig. 1D. For this purpose, cells were incubated with EdU for 30 min just before an acute treatment with high (200 μm) cisplatin concentrations (Fig. 1E). Acute treatment conditions were more appropriate for kinetic analysis of cisplatin adduct formation and decay than long‐term, low concentration treatments and were therefore preferred in subsequent experiments.

Figure 1F shows that after 1 h incubation with cisplatin only a marginal increase in CDA formation was detected in cells, irrespectively of cell cycle phase, while a clear increase was evident at 3 h. This reflects continuing, biphasic CDA formation even after removal of cisplatin from the growth medium—evidently from compound trapped inside the cells. At later times a rapid reduction in CDAs became obvious reflecting, on the one hand, depletion of trapped, free cisplatin and, on the other hand, CDA removal from DNA by cellular repair systems, such as nucleotide excision repair. The kinetic result in Fig. 1F is in line with previous cisplatin‐ICLs measurements obtained using the alkaline comet assay [24], validating R‐C18 as a representative marker for other types of CDAs.

Figure 1G shows the cell‐cycle‐specific analysis of CDAs using the gates shown in Fig. 1D. EdU+ cells represent S‐phase cells and EdU‐ cells can then easily be assigned using the DAPI signal to G_1_ or G_2_ phase. A clear cell‐cycle‐specific increase is observed at 3 and 6 h after treatment, with stronger signals observed in G_2_ phase cells, as compared to S or G_1_ cells. We attribute this increase mainly to the higher DNA content of G_2−_phase cells over G_1_ or S‐phase cells. Results obtained at 12 and 24 h could not be reliably analyzed in a cell cycle‐specific manner, as progression and redistribution of cells through the cell cycle rendered EdU signal analysis uninformative. Based on these results we conclude that the formation of CDAs including ICLs shows no marked cell cycle specificity. Similar experiments have been published by our group with A549 cells showing the feasibility of detection, although cell‐cycle‐specific analysis was not included in that study [40].

### Cisplatin induces DSBs specifically in S‐phase of the cell cycle

**Fig. 2:**
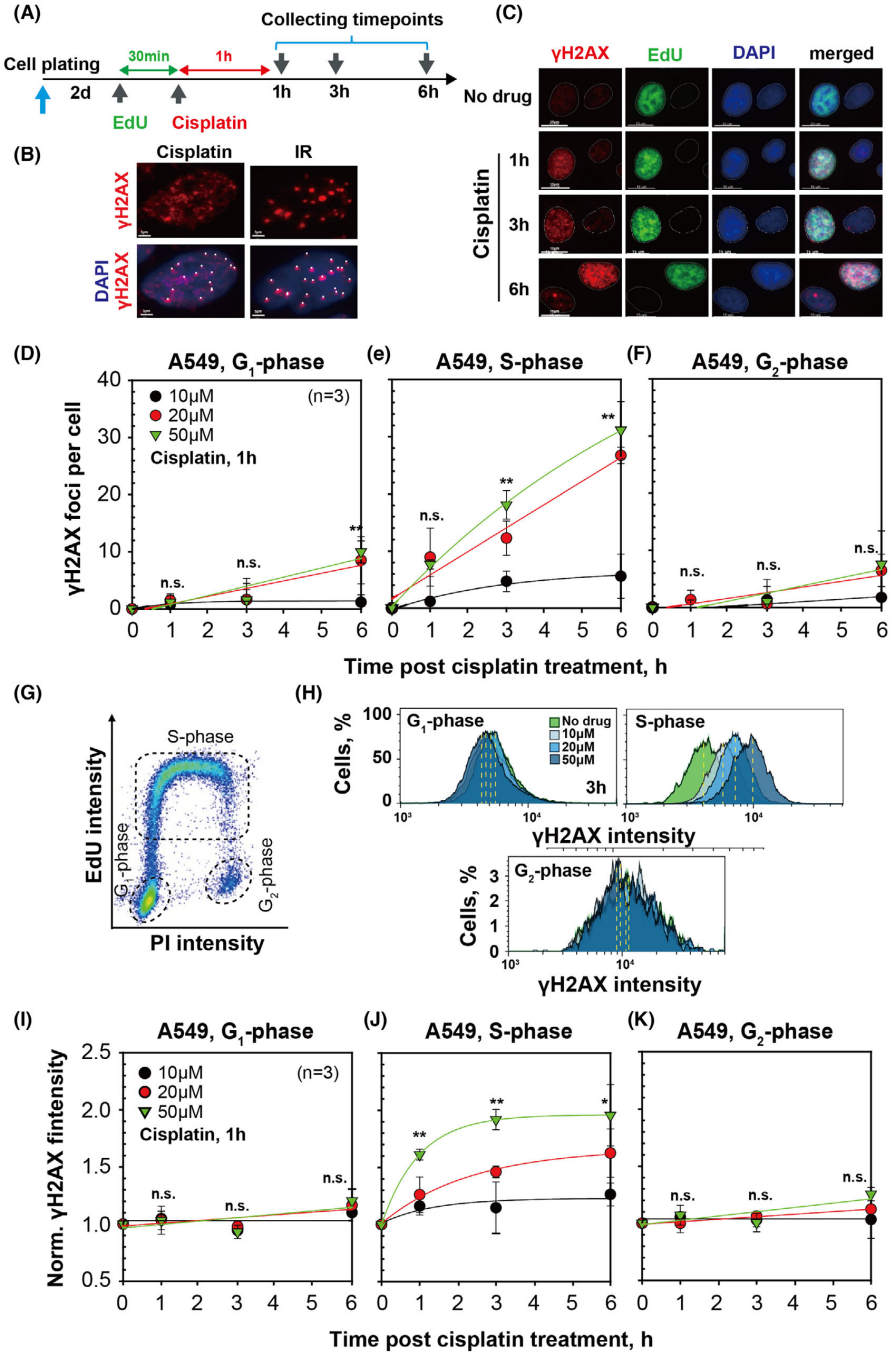
Cisplatin induces double‐strand breaks preferably in S phase of the cell cycle. (A) Schematic of protocol for EdU labeling and cisplatin treatment; (B) Representative images of γH2AX foci induced by cisplatin or IR (ionizing radiation), Scale bar: 3 μm. Dots represent foci identified by the software program; (C) Representative images of γH2AX foci in A549 cells after exposure to 50 μm cisplatin. Scale bar: 15 μm; (D–F) Effects of cisplatin on γH2AX foci induction in A549 cells at the G_1_ (D), S (E), and G_2_ (F) phases of the cell cycle; (G) Representative histogram and dot plots obtained by FC (flow cytometry); (H) FC‐based analysis of γH2AX in A549 cells; histogram shown are representative of three independent experiments with similar results; (I–K) Effects of cisplatin on γH2AX signal intensity in A549 cells at the G_1_ (I), S (J), and G_2_ (K) phases of the cell cycle. Intensity data were normalized to the value measured in untreated controls. Data represent mean ± SD calculated from three independent experiments. Statistical analysis was performed between the no drug group and the 50 μm treatment group. The *P*‐value was calculated using the two‐tailed Student's test. *P < 0.05, **P < 0.01. n.s, nonsignificant.

It is thought that conflicts arising during S‐phase between progressing DNA replication forks and ICLs lead to the induction of lethal DSBs. To investigate this in different phases of the cell cycle, we employed IF to detect γH2AX, a histone variant forming as a consequence of DDR that is widely used as an indicator of DSB formation. Exponentially growing A549 and H460 cells (2 d) were labeled with EdU and treated for 1 h with cisplatin at concentrations between 10 and 50 μm (Fig. 2A). Figure 2B (left) shows that somewhat diffuse γH2AX foci developed in some cell nuclei, with many also showing a relatively high background. This picture differs from the distinct γH2AX foci detected after exposure to IR that are rarely accompanied by a high background (Fig. 2B, right).

Figure 2C shows examples of signals from cells that served as input in the QIBC quantification and Fig. 2D–F the kinetics of γH2AX foci formation in G_1_‐, S‐ and G_2_‐phase obtained in this way. While foci were detectable in all phases examined, it is evident that they were more numerous in S‐phase cells, particularly in the 50 μm treatment group, where the number of foci at 6 h post‐exposure was three times larger than at 1 h (Fig. 2E). Similar results were obtained in H460 cells exposed to 50 μm cisplatin, with a rapid and near‐peak induction in S‐phase and a much more gradual increase in G_1_‐ and G_2_‐phase (Fig. S1A–C). Thus, cisplatin induces faster, markedly more DSBs in S‐phase cells as compared to G_1_ or G_2_ phase cells (Fig. 2D,F, and Fig. S1A–C).

The partly diffuse appearance of γH2AX foci in cisplatin‐treated cells limited scoring accuracy and led us to extend our analysis with a flow cytometry‐based (FC) method that allows quantification of the integrated γH2AX signal per cell in different phases of the cell cycle, as shown in Fig. 2G. Cisplatin‐treated S‐phase cells showed a significant increase in γH2AX signal intensity, while cells of G_1_ or G_2_ phase showed practically no increase (Fig. 2H). Figure 2I–K show a normalized version of the set of times and concentrations analyzed in this experiment. We conclude that DSB formation in cisplatin‐treated cells is an event almost exclusively occurring during S‐phase, as a result of collision of DNA replication forks with ICLs. Indeed, it is likely that the γH2AX signal and foci detected in G_1_‐ or G_2_‐phase in the experiment above actually reflect very early or very late S‐phase cells, and that DSBs actually fail to form in G_1_‐ or G_2_ phase of the cell cycle. More work is required to conclusively address this point.

**Fig. 3:**
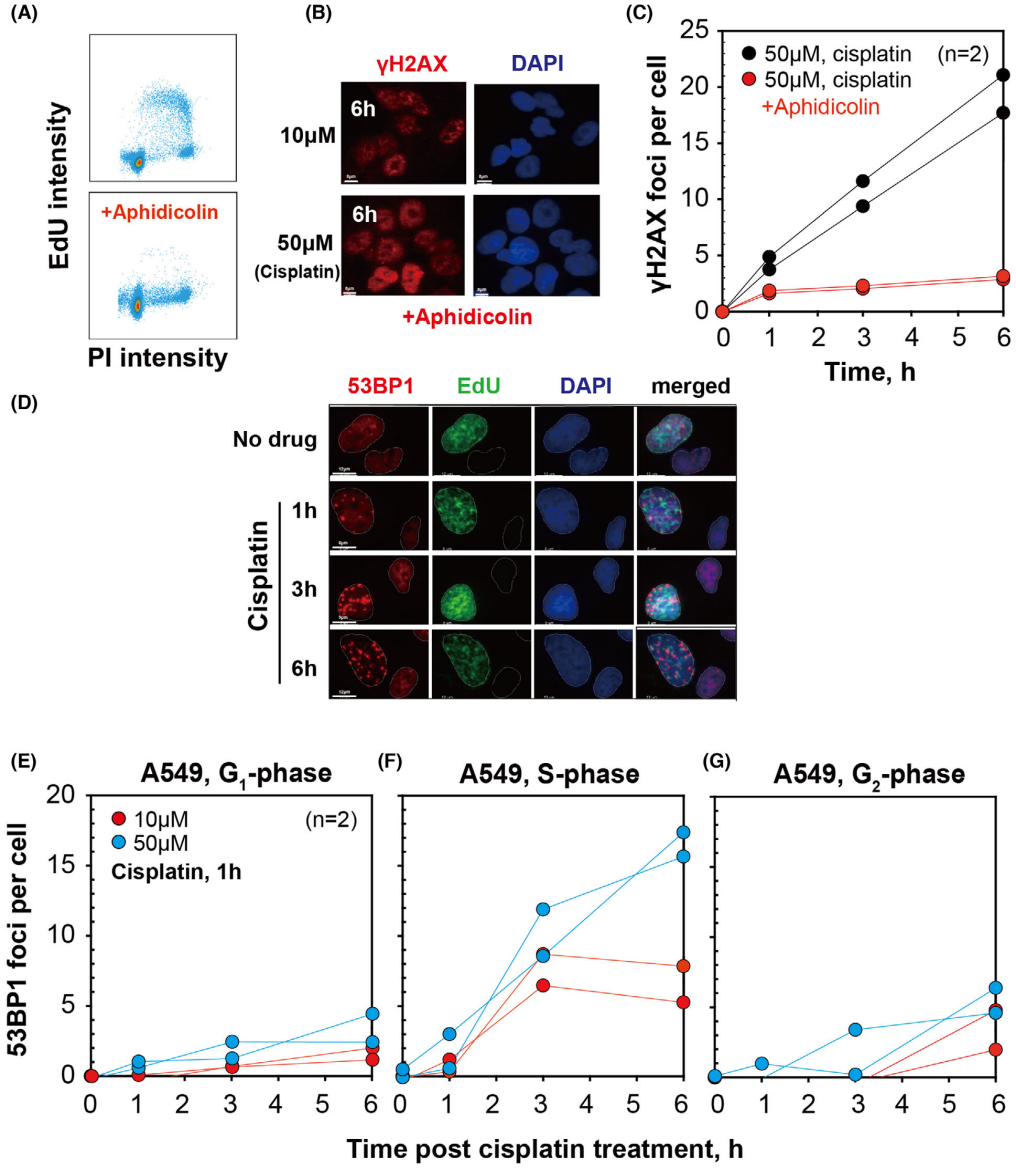
Aphidicolin abrogates γH2AX foci induction in cells exposed to cisplatin. (A) Representative histogram of EdU signal following 1 μm aphidicolin treatment; (B) Representative images of cisplatin‐induced γH2AX foci in A549 cells after aphidicolin treatment. Scale bar: 8 μm; (C) Effects of 50 μm cisplatin combined with or without aphidicolin on γH2AX foci induction in A549 cells; (D) Representative images of 53BP1 foci in A549 cells following 50 μm cisplatin treatment. Scale bar: 12 μm; (E–G) Quantification of 53BP1 foci in cisplatin‐treated A549 cells at the G_1_ (E), S (F), and G_2_ (G) phases of the cell cycle. Each data point represents an independent experiment.

For further evidence for the role of DNA replication in DSB induction in cisplatin‐treated cells, we examined the short‐term effect of aphidicolin, a DNA polymerase inhibitor. Figure 3A shows that aphidicolin completely suppressed EdU incorporation, confirming strong inhibition of DNA replication. Strikingly, treatment with cisplatin in the presence of aphidicolin failed to induce DSBs (Fig. 3B,C), confirming the requirement for active DNA replication for the generation of DSBs.

We also quantitated 53BP1 foci, an alternative marker for DSBs, under similar experimental conditions as for γH2AX. Compared to γH2AX, cells exposed to cisplatin formed well‐defined 53BP1 foci. The results in Fig. 3D–G confirm that DSBs form primarily in S phase cells.

### CDAs leave unchanged repair of IR‐induced DSBs

It is conceivable that when cisplatin treatment is combined with IR to treat cancer, CDAs present in the genome interfere with the repair of IR‐induced DSBs, even in G_1_‐ or G_2_ phase. Additional interference and complications may arise in S‐phase cells, where, in addition to CDAs, also replication‐associated DSBs are expected to form during the processing of IR‐induced DSBs. Thus far, definitive answers to these questions are lacking despite their relevance to the mechanistic understanding of cisplatin–radiation interactions.

**Fig. 4:**
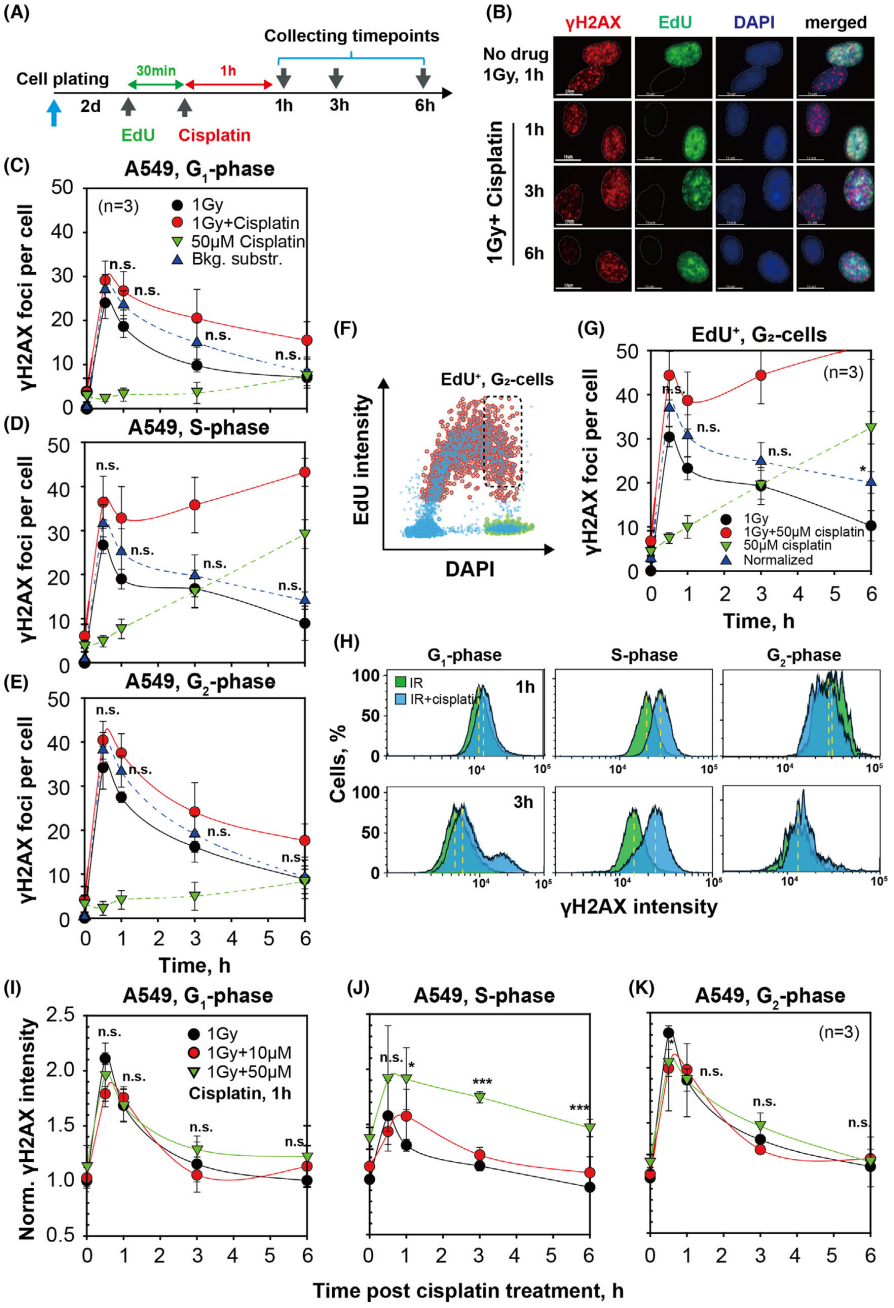
Cisplatin enhances the IR‐induced double‐strand breaks level without affecting repair. (A) Schematic of protocol for EdU labeling, cisplatin treatment, and radiation exposure; (B) Representative images of γH2AX foci following 1Gy IR (ionizing radiation) and 50 μm cisplatin in A549 cells. Scale bar: 15 μm; (C–E) Effects of cisplatin on IR‐induced γH2AX foci kinetics in A549 cells at the G_1_ (C), S (D), and G_2_ (E) phases of the cell cycle; (F) Representative histogram and dot plots obtained from QIBC (quantitative image based cytometry) showing EdU^+^ G_2_ compartment gating (boxed area); (G) Effects of cisplatin on IR‐induced γH2AX foci kinetics in EdU^+^ G_2_ A549 cells; (H) FC (flow cytometry)‐based analysis of γH2AX in A549 cells; histogram shown are representative of three independent experiments with similar results; (I‐K) Effects of cisplatin combined with IR on γH2AX signal intensity in A549 cells at the G_1_ (I), S (J), and G_2_ (K) phases of the cell cycle. Intensity data were normalized to the value measured in non‐irradiated controls. Data represent mean ± SD calculated from three independent experiments. Dashed lines represent data from non‐IR groups. Statistical analysis was performed between background subtracted and radiation alone groups, the *P*‐value was calculated using the two‐tailed Student's test, *P < 0.05, ***P < 0.001. n.s, nonsignificant.

The experimental protocol developed to address these questions is outlined in Fig. 4A: Exponentially growing (2 d) cells were sequentially incubated with EdU, then with cisplatin for 1 h, and immediately thereafter exposed to 1 Gy of IR. γH2AX foci formation was analyzed in these cells as a function of time thereafter in a cell cycle‐dependent manner. As repeatedly demonstrated [18, 35], γH2AX foci formed rapidly in irradiated cells and peaked at 30 min; 50% of them were resolved at 6 h, documenting repair of IR‐induced DSBs. This repair pattern was observed across all three cell cycle phases analyzed after IR alone (Fig. 4B–E).

When radiation was combined with 50 μm cisplatin, maximum γH2AX foci levels increased somewhat across all cell cycle phases. The most pronounced effect was observed in cells undergoing replication, but in all phases examined this increase only occasionally reached statistical significance (Fig. 4C–E). Since this increase derives from the independent induction of γH2AX foci in cisplatin‐treated cells, we reanalyzed the results by subtracting foci counts from cisplatin alone treatment at each time point. These background‐subtracted results demonstrated that cisplatin exerts a relatively small inhibitory effect on the repair of IR‐induced DSBs in all phases of the cell cycle examined (Fig. 4C–E). We conclude that the presence of CDAs in the genome only slightly interferes with the repair of IR‐induced DSBs.

Because both cisplatin treated and irradiated cells continue to progress through the cell cycle, we inquired whether repair defects become apparent when cells treated in S‐phase progress into the G_2_‐phase of the cell cycle, where HR is fully active. Such analysis is possible with the treatment protocols employed here, because cells from S‐phase can be identified in G_2_‐phase by virtue of their EdU^+^ staining (Fig. 4F). Figure 4G shows that under these conditions the effect of cisplatin alone was more pronounced in the sense that it produced more DSBs owing to the time elapsed since the beginning of cisplatin treatment. Interestingly, in this case, subtraction of these foci from those deriving from IR uncovered reduced overall repair for IR‐induced DSBs at levels that started reaching statistical significance, although the half time of repair was not affected (Fig. 4G).

We also evaluated the kinetics of IR‐induced DSBs in cells that carry CDAs using FC. Figure 4H shows for cells exposed to IR alone that DSBs are detectable and that DSB repair is active in G_1_–S‐ and G_2_‐phase of the cell cycle. After exposure to cisplatin, a stronger γH2AX signal is detected, specifically in S‐phase, owing to the induction of cisplatin generated DSBs. Normalization to background (rather than subtraction) of the latter signal, and compilation of results from several experiments carried out at different treatment times, generated the results summarized in Fig. 4I–K, which again show that CDAs have a clear effect on γH2AX signal levels, but less effect on the half time of IR‐induced DSB repair.

The data above primarily reflect the cellular response to low‐dose IR (1 Gy). At high IR doses, however, cNHEJ is considered to take over HR as the predominant DSB repair pathway [35], and the likelihood of its interaction with CDA may increase. We therefore investigated whether cisplatin inhibits the repair of DSBs induced by higher IR doses. Since the γH2AX signal saturates at high IR doses compromising foci analysis, we employed PFGE—a technique that directly detects physical DNA damage in the genome. Results depicted in Fig. S1E,F show that approximately 90% of the damage induced by 20 Gy IR was repaired within the first 30 min. Notably, the presence of CDA in the DNA failed to alter this repair kinetics (Fig. S1E,F).

Moreover, markers reflecting DNA end resection (RPA70) and HR activity (RAD51) were followed over time in cisplatin‐treated A549 cells using IF protocols similar to those for γH2AX. Notably, the formation of CDAs did not affect the initiation and progression of DNA end resection in these cells throughout the cell cycle (Fig. S2B–D). A moderate delay in RAD51 foci formation and reduced resolution were observed in S‐ and G_2_‐phase A549 and H460 cells treated with cisplatin and exposed to IR (Fig. S3B,C,E,F). These data in aggregate suggest that CDAs fail to generate a global suppressive effect on the repair of IR‐induced DSBs. The effects observed on HR are interesting and warrant further investigation.

### Only long‐term cisplatin exposure mediates radiosensitization

The results of the previous section suggest that cisplatin may not be acting as a classical radiosensitizer that interferes with the repair of radiation‐induced DSBs. However, cisplatin radiosensitization is documented in several experimental settings and is supported by clinical data [3, 18, 45]. We explored therefore the possibility of alternative mechanisms of radiosensitization.

**Fig. 5:**
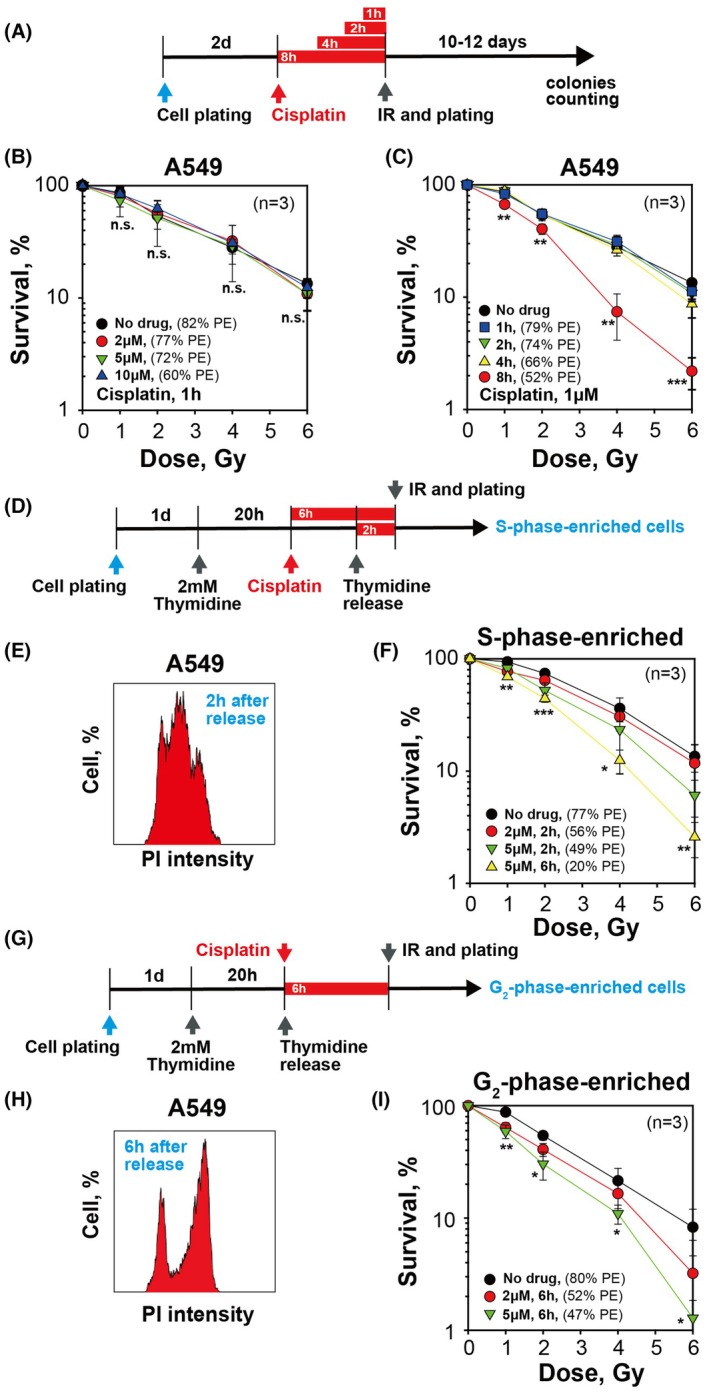
Long‐term treatment of cisplatin before IR mediates radiosensitization on lung cancer cells. (A) Schematic of treatment protocol used in (B and C); (B) Clonogenic survival in A549 cells demonstrating radiosensitization after 1 h incubation with 2–10 μm of cisplatin; (C) Radiosensitization assessed in (B) for A549 cells after 1–8 h pre‐IR (ionizing radiation) incubation with 1 μm cisplatin; (D) Schematic of cell cycle synchronization and treatment protocol used in (F); (E) Representative histogram of cell cycle distribution at the time of irradiation; (F) Clonogenic survival of A549 cells enriched in the S phase, showing radiosensitization after 2 or 6 h incubation with 2 or 5 μm of cisplatin; (G) Schematic illustration of treatment protocol used in (I); (H) Representative histogram of cell cycle distribution at the time of irradiation; (I) Radiosensitization assessed as in (F) for A549 cells enriched in the G_2_ phase after 6 h incubation with varying concentrations of cisplatin. Data represent mean ± SD calculated from three independent experiments. Statistical analysis was performed between no drug and cisplatin‐treated groups (10 μm group in B; 8 h group in C; 5 μm, 6 h groups in F and I); the *P*‐value was calculated using the two‐tailed Student's test. *P < 0.05, **P < 0.01, ***P < 0.001. n.s, nonsignificant. PI (propidium iodide); PE (plating efficiency).

Figure 5B shows that treatment of A549 cells with cisplatin (2–10 μm) for 1 h before irradiation has no radiosensitizing effect, when studied using colony formation as endpoint, while higher concentrations or treatment times were strongly cytotoxic. In the clinic, cisplatin is administered over several cycles concurrently with radiotherapy using different regimens depending on the tumor type. Thus, cells with CDAs they sustained hours or days earlier might be exposed to radiation. To examine whether radiosensitization may occur under these conditions, we treated A549 cells with 1 μm cisplatin for 1, 2, 4, and 8 h before exposing to radiation to measure cell survival (Fig. 5A). The results summarized in Fig. 5C show that while pre‐incubation up to 6 h with cisplatin failed to radiosensitize, treatment for 8 h caused pronounced radiosensitization. Notably, the degree of radiosensitization measured at 8 h pre‐incubation is the maximum achievable in this type of experiment, as it remains practically unchanged even after 48 h pre‐incubation (Fig. S4A). Even an increase in cisplatin concentration by an order of magnitude (10 μm) fails to further enhance this effect (Fig. S4B). Similar responses are also found in H460 cells analyzed using identical protocols (Fig. S4C,D).

But what is the mechanistic base of the observation that radiosensitization reached maximal levels after 8 h of pre‐exposure at all cisplatin concentrations tested? Two not mutually exclusive models can be developed. The first assumes that cisplatin‐treated cells reach a phase in the cell cycle that is characterized by increased radiosensitivity. There are two stages in the cell cycle where radiosensitivity to killing reaches a maximum: the G_1_/S and G_2_/M borders. We hence employed one and two‐parameter (H3pS10/PI) FC analysis to assess the cell cycle distribution in cells at the time of IR exposure (8 h). The single parameter FC data shown in Fig. S5A,B show no differences in the distribution of cells in the cell cycle able to explain the observed radiosensitizing effect. This conclusion remains valid even after inspection of results on the effects of cisplatin on MI obtained using two‐parameter FC analysis (Fig. S5C,D). Although strong inhibition of the MI is observed after incubation to cisplatin at concentrations above 10 μm (Fig. S5C,D), the low (~2%) representation of mitotic cells in the population precludes a measurable contribution to the overall radiosensitization.

Alternatively, it may be postulated that cisplatin DNA lesions generated during replication evolve within 8 h, most likely in G_2_‐phase, to lesions that interfere with the repair of IR‐induced DSBs. The results in Fig. 4G provide some support for this postulate. To begin addressing this possibility, we initiated experiments with cells enriched in specific phases of the cell cycle.

To this end, we used 2 mm thymidine for 20 h to arrest cells at the G_1_/S boundary [44]. Upon release into thymidine‐free fresh growth medium (Figs 5D and S6), cells entered S phase and had at 2 h the DNA content distribution shown in Fig. 5E. Figure 5F shows their radiosensitivity at this point. The same set of cells was exposed to 2 or 5 μm cisplatin according to two protocols. First, cells were exposed to cisplatin for 2 h after release from the thymidine block and were subsequently irradiated and plated to measure survival. It is evident that this treatment generated marked radiosensitization, particularly at the higher concentration of 5 μm cisplatin (Fig. 5F). In a second treatment group the 2 h of incubation with cisplatin after release from the thymidine block was enhanced by an additional 6 h treatment during the thymidine block. It is evident that even stronger radiosensitization could be detected under these conditions (Fig. 5F).

In a similar experiment, cells were treated for 6 h with 2 or 5 μm cisplatin immediately after release from the thymidine block (Fig. 5G) and were irradiated as highly enriched, G_2_‐phase populations (Fig. 5H). It is evident that this prolongation of cisplatin treatment after thymidine block release further enhanced radiosensitization (Fig. 5I). Note that progression of cisplatin‐treated cells through the cycle was practically identical to that of untreated controls (Fig. S6).

The above results uncover a unique point of radiosensitization by cisplatin lying around the G_1_/S border—the phase in the cell cycle when cells with the maximum radiosensitization first saw cisplatin. Since intrinsically, cells are mostly radiosensitive right at the G_1_/S border and are expected to have gained resistance 2 h after release from the thymidine block, the results suggest that cisplatin lesions induced in the DNA at the G_1_/S border are capable of interacting with IR‐induced DSBs and inhibiting their repair, causing radiosensitization. Because this likely pertains to a small subpopulation of cells, it remains marginally detectable in the kinetics shown in Fig. 4. This response may also be related to the absence of replicated DNA at this stage of the cell cycle that is required for HR.

**Fig. 6:**
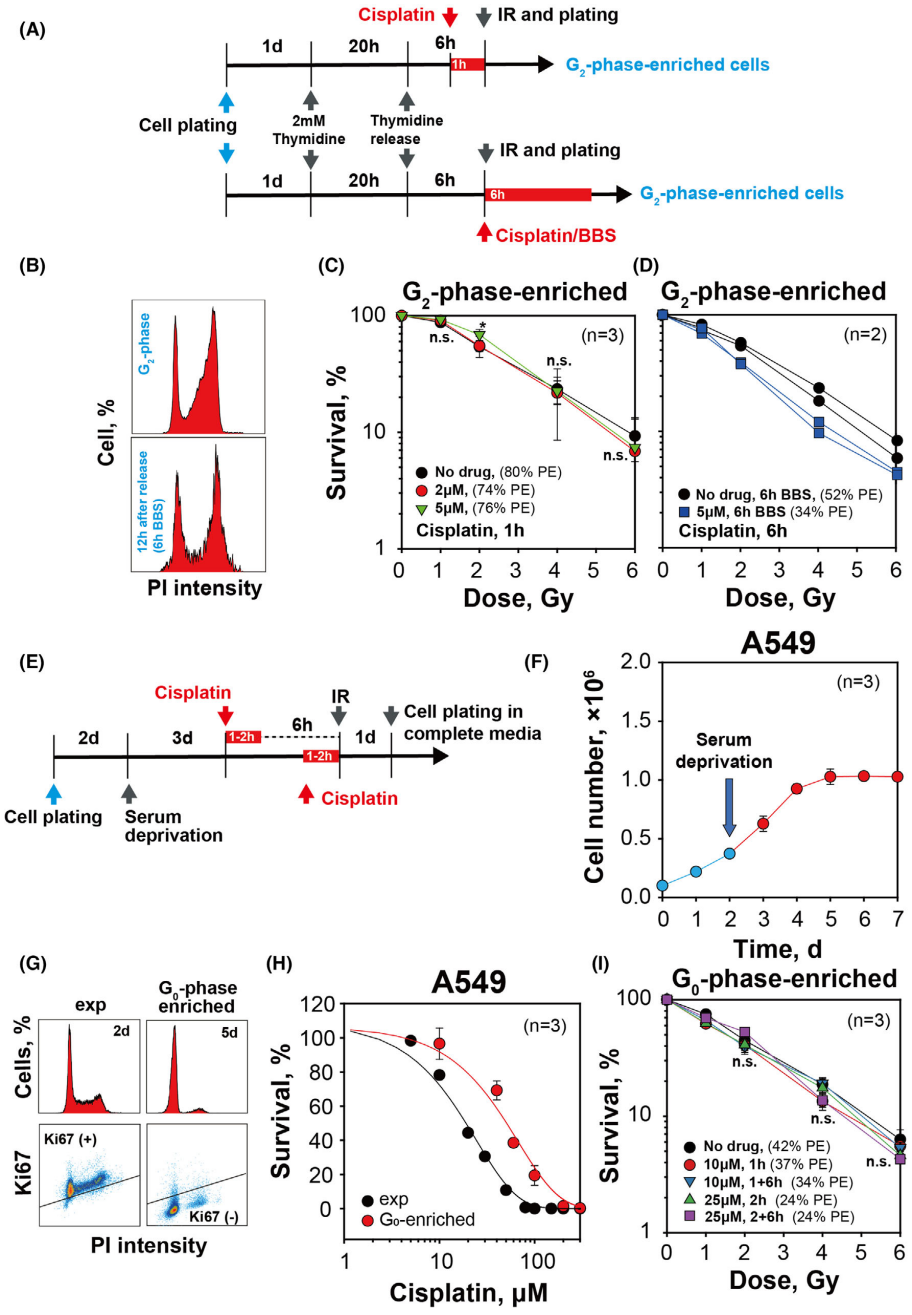
Non‐replicative status reduces cell radiosensitization to cisplatin. (A) Schematic of cell cycle synchronization and treatment protocol used in (C and D); (B) Representative histogram of cell cycle distribution after incubation with BBS (balanced salt solution) for 6 h; (C) Clonogenic survival of G_2_‐enriched A549 cells showing radiosensitization after 1 h incubation with 2 or 5 μm of cisplatin; (D) Radiosensitization assessed as in (C) for G_2_‐enriched A549 cells after 6 h incubation with 5 μm of cisplatin and BBS; (E) Schematic of serum deprivation and treatment protocol used in (I); (F) Cell number over time in culture following serum deprivation; (G) Representative histogram of cell cycle distribution and Ki‐67 signal after serum deprivation; (H) Comparison of cisplatin cytotoxicity in serum deprived and exponentially growing cells; (I) Radiosensitization of A549 cells enriched in the G_0_ phase treated with 10 or 25 μm cisplatin (1–2 h) with/without additional 6 h recovery before IR (ionizing radiation). Data represent mean ± SD calculated from three independent experiments (except for the data in Figure D, where each data point represents an independent experiment). Statistical analysis was performed between no drug and cisplatin‐treated groups (5 μm groups in C; 25 μm, 2 + 6 h group in I); the *P*‐value was calculated using the two‐tailed Student's test. *P < 0.05. n.s, nonsignificant. PI (propidium iodide); PE (plating efficiency).

We also investigated radiosensitization in cells exposed to 2 or 5 μm cisplatin in G_2_‐phase. In this experiment, one set of cells was exposed for 1 h at the end of this 6 h period of cell cycle progression after the thymidine block (Fig. 6B) and was subsequently irradiated (Fig. 6A upper scheme). Strikingly, no radiosensitization was observed (Fig. 6C). To investigate whether this is due to the short treatment duration, in a second set of cells we extended treatment with 2 or 5 μm cisplatin to 6 h (Fig. 6A lower panel). To reduce the rate of cell division and investigate more specifically G_2_‐effects, cells were transferred to balanced salt solution (BSS) [41]. Even after prolonged treatment at the high cisplatin concentration, only a moderate degree of radiosensitization was observed (Fig. 6D). The above results collectively suggest that for radiosensitization to occur, exposures to cisplatin during S‐phase and progression through the cell cycle are required.

To further confirm this postulate, we evaluated cisplatin radiosensitization by colony formation in a non‐replicating cell system. Using A549 cells, we generated G_0_ cell populations by growing cells, first for 2 days in regular growth medium and transferring them subsequently for 3 days to serum‐free medium (Fig. 6E) [42]. Serum deprivation led to an accumulation of cells with G_1_‐phase DNA content (Fig. 6F) and the cessation of cell proliferation (Fig. 6F). The proliferation marker Ki‐67 shows that cells actually accumulated in G_0_ (Fig. 6G). These G_0_‐enriched cells exhibited resistance to cisplatin when given continuously for 24 h before plating (Fig. 6H). To measure cisplatin radiosensitization the treatment protocol in Fig. 6E was applied that included a 1–2 h treatment with 10 or 25 μm cisplatin given either 6 h before, or immediately before irradiation. After irradiation, cells were kept for 24 h in serum‐free medium to delay re‐entering into S‐phase and to avoid the S‐phase‐specific effects described above. Notably, under these conditions, no radiosensitization by cisplatin could be detected (Fig. 6I). Collectively, our findings suggest that cell cycle progression from S to G_2_ phase during cisplatin treatment is a fundamental requirement for cisplatin radiosensitization.

## Discussion

### Similar yields of CDA in different cell cycle phases

To study the mechanistic underpinning of cisplatin–radiation interactions in a cell cycle‐specific manner, it is important, as a first step, to consider possible fluctuations in the induction of relevant lesions. For radiation, it has long been established that the yield of DSBs per unit of DNA mass is constant throughout the cell cycle. Much less work is available along these lines with cisplatin.

Over the past 40 years, various methods have attempted to characterize CDA, such as spectroscopic procedures [46], plasma mass spectrometry [47], or isotope labeling techniques [48, 49]. However, these techniques either cannot distinguish specific lesions or do not permit single‐cell resolution. Subsequently, antibody‐based immunoassays were developed to quantify CDA at the single‐cell level [38, 50, 51], including the development by our group of a series of monoclonal antibodies (R‐B3, R‐C7, R‐C18) with defined specificities for CDA [38]. These techniques have enabled the study of the kinetics of CDA formation and removal in various cancer cell lines, animal tissues, and patient samples [38, 46, 47, 48, 49, 50, 51]. Nevertheless, the cell cycle‐related characteristics of CDA remained unexplored. Here, using the R‐C18 antibody and co‐staining of DNA with DAPI and the S‐phase fraction with EdU, we demonstrate that the yields of CDA are similar throughout the cell cycle. These results facilitate the analysis of the molecular mechanisms of cisplatin‐related cytotoxicity and radiosensitization.

### CDA cause DSBs specifically during S‐phase

Despite the similar yields of CDA throughout the cell cycle, the induction of DSBs in cisplatin‐treated cells is strongly cell cycle dependent and specific for cells in S‐phase. Our observations along these lines agree with previous work that used flow cytometry analysis similar to that employed here to show S‐phase specific induction of γH2AX signal [24], and no γH2AX signal in unstimulated cisplatin‐treated human peripheral blood lymphocytes [24, 52]. The S‐phase‐dependent induction of DSBs by cisplatin is mainly a consequence of replication conflicts with ICLs. Indeed, previous studies with various ICL‐inducing agents including nitrogen mustard (HN2), mitomycin C (MMC), and photoactivated psoralens have demonstrated that ICL‐induced DSB formation requires cell cycle progression into S phase [53, 54, 55] and is absent in non‐cycling populations [53, 54, 55]. However, it should be noted that intrastrand cisplatin adducts also have potential for DNA replication fork disruption and therefore for DSB induction, albeit at lower levels.

### Effects of cisplatin on DSB repair

It is still debated whether cisplatin affects DSB repair. We have reported previously that DSB rejoining, as measured with the constant‐field gel electrophoresis assay, is only marginally reduced upon cisplatin treatment [52]. Our γH2AX analysis also shows that the presence of cisplatin adducts in the DNA fails to immediately compromise the repair of IR‐induced DSB [19]. These earlier results together with the results reported in the present study suggest that CDAs‐induced inhibition of IR‐generated DSBs may not be the main determinant of cisplatin radiosensitization.

Other studies, however, implicated c‐NHEJ inhibition in cisplatin‐mediated radiosensitization. This conclusion was drawn from the absence of sensitization in Ku80‐ or DNA‐PK‐deficient cell models. The specific requirements of radiosensitization uncovered in these experiments suggest caution when comparing with our results [11, 56].

It is relevant to point out that in the specifically constructed *in vitro* systems of these studies, cisplatinated DSBs suppressed DNA‐PK‐dependent end‐joining [12, 13]. Indeed, when 1,2‐d(GpG) adducts were incorporated at varying sites relative to DNA terminus to assess DNA‐PK kinase activity and Ku translocation efficiency, a spatial relationship was revealed in the inhibition of DNA‐PK by CDAs: adducts located 6–20 bp from the terminus impaired both Ku translocation and DNA‐PK activation, which completely blocked c‐NHEJ *in vitro*. In contrast, CDA positioned ≥ 60 bp from the terminus showed no effect on DNA‐PK activation [11, 14, 17].

The consequences of cisplatin‐adducted DSBs were further investigated using host‐cell reactivation assays. When cells were pretreated with cisplatin prior to transfection with undamaged reporter plasmids, no reduction in NHEJ efficiency was observed. While transfection of untreated cells with reporter plasmids containing cisplatin lesions positioned 6‐bp from DSB termini resulted in significant c‐NHEJ impairment [13]. This effect of cisplatin on NHEJ was confirmed by Diggle et al. [12] in another cell‐free assay using adduct levels commensurate with clinical doses of concurrent chemoradiation. PFGE is a commonly used technique for investigating the engagement of c‐NHEJ in DSB processing. In this assay, the repair kinetics of DSB repair following high‐dose IR exposures (10–20 Gy) are mainly determined by the activity of c‐NHEJ [35, 43]. Notably, using this assay, we failed to detect changes in DSB rejoining in CDA‐containing DNA.

Collectively, the above evidence supports the notion that cisplatin adducts inhibit c‐NHEJ‐mediated DSB repair only when present in the immediate vicinity of the DNA end. However, based on measured data and theoretical calculations, if adducts are randomly distributed, only 0.5%–1% of adducts would localize within 10 bp of a DSB at clinically relevant doses of cisplatin and radiation, and in ~0.1%–0.2% of cases would experience impaired end‐joining [12, 57]. Thus, the overall contribution is expected to be rather low and therefore undetectable in the type of experiments we performed.

Notably, recent work reported that at CDA sites, X‐rays produce increased numbers of secondary electrons and clustered DNA damage, which may affect repair and cause radiosensitization [58, 59, 60, 61]. Despite the expected rather low incidence of such events as well, the possibility of altering DSB lesion complexity as a consequence of CPA presence is intriguing and requires further rigorous experimental consideration.

There are reports that repair of IR‐induced DNA strand breaks measured by FADU (fluorometric analysis of DNA unwinding) can be altered by a high concentration (10 μg·mL^−1^) of cisplatin, with more residual damage detected [62]. Also, another study [18] reported that combined cisplatin–radiation treatments cause increased and persistent γH2AX foci at 24 h. Although our results are in principle in line with these reports, it is important to note that these studies focused on the composite effects of the combined treatment and used assays in which ICLs may directly affect the principle of DSB detection (unwinding) and also be sensitive to cell cycle disruptions. Our study was designed to measure in a cell‐cycle‐dependent manner cisplatin's direct modulation of IR‐induced DSB repair, through proper normalization for background damage caused by cisplatin alone. Our results help therefore in the mechanistic analysis of the combined effects in a strictly cell‐cycle‐dependent manner.

Additional improvements in our study include the fact that we examined γH2AX foci using QIBC to detect thousands of cells per analysis point and included a highly specific analysis in S‐phase cells through the use of EdU labelling. When combined with low‐dose radiation (1–2 Gy), cisplatin in our study increased somewhat IR‐induced DSB levels without significantly attenuating DSB repair kinetics.

### Mechanisms of cisplatin radiosensitization

A common assumption in the field is that cisplatin somehow synergizes with IR‐induced DNA damage and enhances radiosensitivity to killing [19, 45, 63, 64]. It has also been reported that cisplatin‐mediated radiosensitization is both time and sequence dependent. Specifically, for radiosensitization, drug exposure must precede irradiation, and sufficient drug action time is required for an effect [18, 65, 66]. Thus, maximal radiosensitization was achieved in A549 and H460 cells when cisplatin was administered 6 h before irradiation, whereas exposures within 4 h, or after irradiation, showed no effect [19, 65].

Our results extend these observations and provide for the first time a mechanistic basis for these time and sequence requirements. They clarify that CDA‐mediated replication fork damage during transition from S to G_2_ phase is a requirement for effects on IR‐induced DSB repair and radiosensitization. Indeed, synchronized cells with sufficient CDA loads exhibited after S‐phase progression markedly enhanced radiosensitivity in G_2_, whereas cells treated post‐synthesis, or while in a quiescent state, showed less or no such response.

Conditions like these are likely to be satisfied during the clinical application of cisplatin, where CDAs were shown to persist at high levels in tumor tissues throughout the chemoradiation cycle. Indeed, their presence correlates positively with the cumulative dose of cisplatin, regardless of the schedules of cisplatin administration employed [67]. Under conventional fractionated RT, DNA of cancer patients accumulates sufficient loads of CDAs before IR, and some tumor cells are likely to fulfill the required transition through S‐phase to achieve radiosensitization. The latter effect is expected to be more pronounced during phases of accelerated repopulation in the tumor, allowing thus cisplatin to continue its effective radiosensitization in cells entering S phase.

From a theoretical perspective, enhancing the spatial interaction probability between IR‐induced DSB and CDA would require higher doses of cisplatin. While cisplatin dose escalation is constrained by hematological and renal toxicity, the advent of stereotactic radiotherapy (SRT) techniques now provides potential for the application of higher IR doses to maximize effect. It is worth noting that upon high‐dose radiation (>5 Gy), NHEJ is considered to take over HR as the predominant repair pathway [35, 68]. Potentially, CDA‐mediated NHEJ inhibition may thereby create a vulnerability that enables cisplatin to potentiate the tumor cell radiation response. Moreover, CDA has been suggested to alter the remodeling of higher‐order chromatin structure around DSB sites [69, 70, 71] that may inhibit repair, causing thus additional, beneficial radiosensitization.

Exploiting the downstream cellular response can also be a strategy to enhance cisplatin–radiation efficacy. Replication‐associated damage generated by cisplatin activates comprehensive DNA damage response (DDR), most notably through ATM/ATR‐mediated cell cycle checkpoint activation [18, 30, 45, 52]. This activation is reflected in a marked increase in ATM/ATR phosphorylation levels [18, 31, 45]. Moreover, abolishing this regulatory safeguard by integrating ATM and ATR inhibitors into chemoradiation regimens is indeed shown to dramatically enhance cisplatin‐mediated radiosensitization [18, 45].

### Connections with the clinical application of cisplatin

Early in 1994, Armstrong et al. [72] explored the association between CDA levels and short‐term tumor response in clinical practice. Bone marrow aspirates from 14 acute leukemia patients receiving chemotherapy were incubated with cisplatin *in vitro*, revealing that aspirates from patients who achieved complete remission had significantly lower adduct levels than those from patients who had remission failures [72]. In a study by Hoebers et al. [73] involving 35 patients with head and neck squamous cell carcinoma receiving cisplatin‐RT, results indicate that patients with higher adduct levels in post‐treatment tumor biopsies had favorable disease‐free survival and overall survival [73]. For patients from whom primary tumor biopsies were almost inaccessible, adduct levels could be assessed testing buccal cells. Multivariate analyses by Van de Vaart et al. [74] suggest that buccal cell CDA levels were an independent prognostic factor for NSCLC patients receiving RT concurrent with daily cisplatin. These studies highlight the clinical significance of monitoring CDA during RT in each individual tumor. Finally, outcomes from the recent DIAMOND trial have shown that a subset of nasopharyngeal carcinoma patients could be exempted from subsequent concurrent cisplatin chemotherapy after cisplatin‐containing induction chemotherapy [75]. This may also support our conclusion that maximal radiosensitization could be achieved when tumor cells accumulate sufficient CDA loads prior to IR.

## Conclusion

The formation of CDA is similar across the cell cycle, yet the resultant DSBs manifest exclusively during S‐phase. Whereas CDAs fail to interfere with repair of IR‐induced DSBs, progression‐dependent repair disruptions cause radiosensitization. Investigating these underlying processes could pave the way for rationally designed, more potent cisplatin–radiation combination therapies.

## Conflict of interest

Martin Stuschke: AstraZeneca (Advisory Board Function, Research and Clinical Trials), Bristol‐Myers Squibb (Advisory Board Function), Sanofi‐Aventis (Advisory Board Function), and Janssen‐Cilag (Advisory Board Function). Eleni Gkika AstraZeneca (Advisory Board Function, Research and Clinical Trials). Other authors declare no conflict of interest.

## Author contributions

YQ and XL: Writing – review and editing, Writing – original draft, Visualization, Methodology, Investigation, Formal analysis, Data curation, Conceptualization. EM and VM: Writing – review and editing, Writing – original draft, Visualization, Validation, Supervision, Methodology, Investigation, Formal analysis, Data curation, Conceptualization. YW and YD: Data curation; Validation. EG, AS and JT: Writing—original draft, Methodology, Formal analysis, Data curation. MS: Supervision, Resources, Project administration, Funding acquisition, Conceptualization. GI: Writing – review and editing, Writing – original draft, Visualization, Validation, Supervision, Software, Resources, Project administration, Funding acquisition, Conceptualization.

## Supporting information


**Figure S1.** Effects of Cisplatin on DSB induction and repair in H460 cells.

**Figure S2.** Cisplatin has no impact on DNA end resection.

**Figure S3.** Cisplatin causes a delay on the formation of RAD51 foci.

**Figure S4.** Assessments of cisplatin‐mediated radiosensitization under different treatment conditions.

**Figure S5.** Effects of cisplatin treatment of the distribution of cells throughout the cell cycle.

**Figure S6.** Cell cycle progression after the release from thymidine block.

**Table S1.** Drugs and antibodies.

## Data Availability

Data will be provided upon reasonable request.
