# Supplementary figures and images for: Cell‐cycle‐specific lesion evolution rather than inhibition of double‐strand‐break repair underpins cisplatin radiosensitization
Source: Mol Oncol. 2026 Mar 19;20(7):1814–34. doi: 10.1002/1878-0261.70240 (PMC13352966; doi:10.1002/1878-0261.70240)

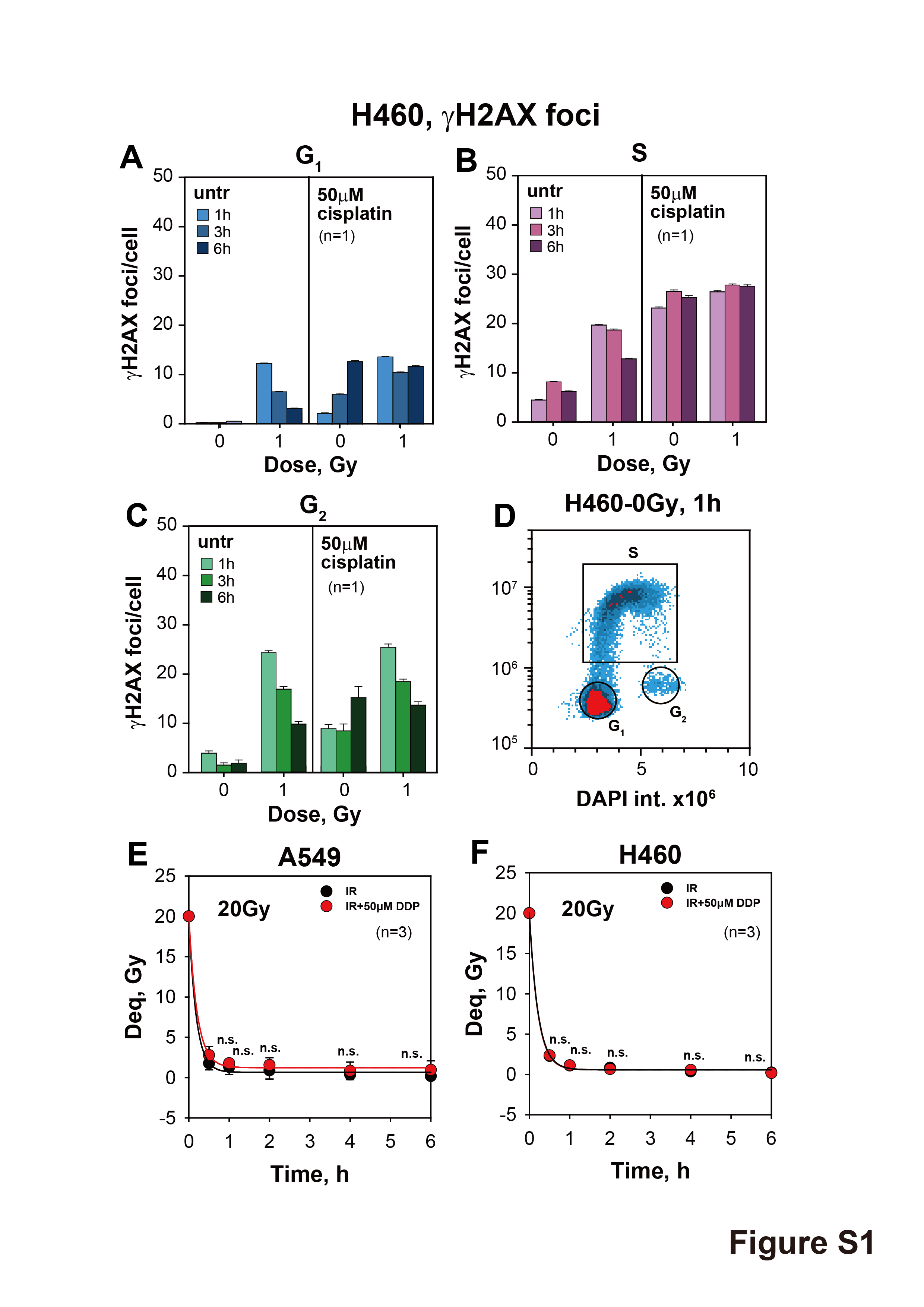

Supplement: Supplementary file 1 — Figure S1. Effects of Cisplatin on DSB induction and repair in H460 cells. [file MOL2-20-1814-s001.jpg]

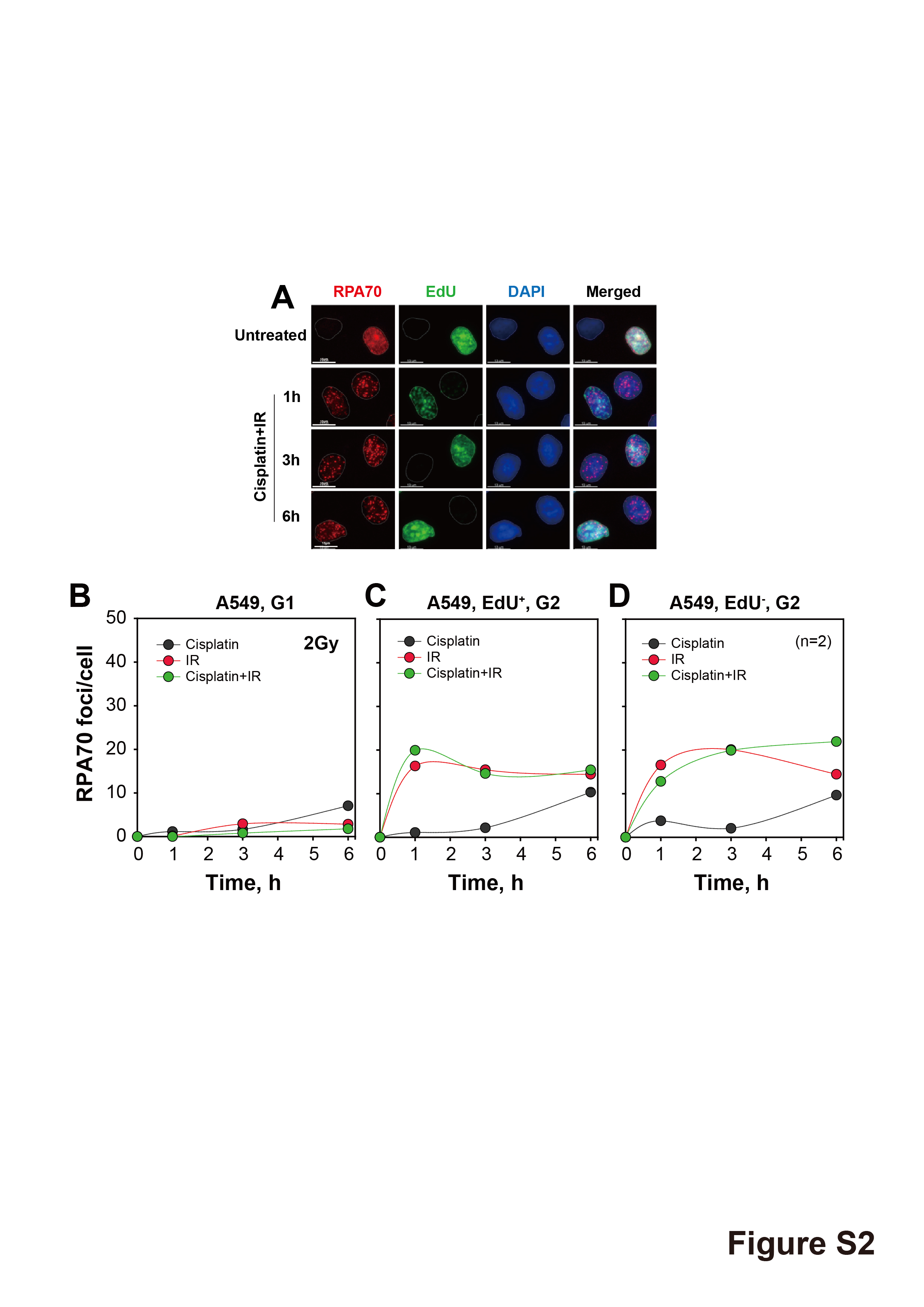

Supplement: Supplementary file 2 — Figure S2. Cisplatin has no impact on DNA end resection. [file MOL2-20-1814-s002.jpg]

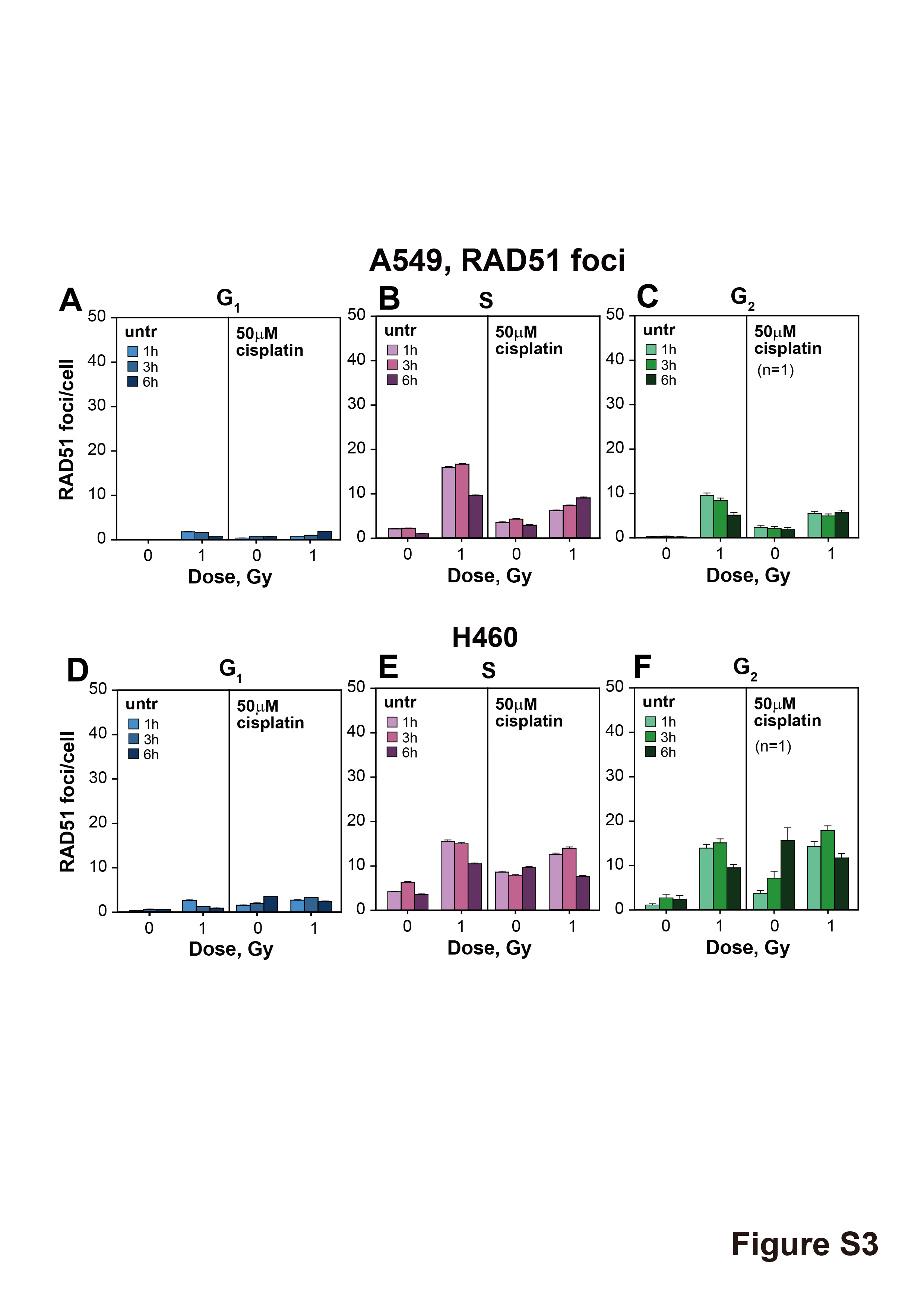

Supplement: Supplementary file 3 — Figure S3. Cisplatin causes a delay on the formation of RAD51 foci. [file MOL2-20-1814-s007.jpg]

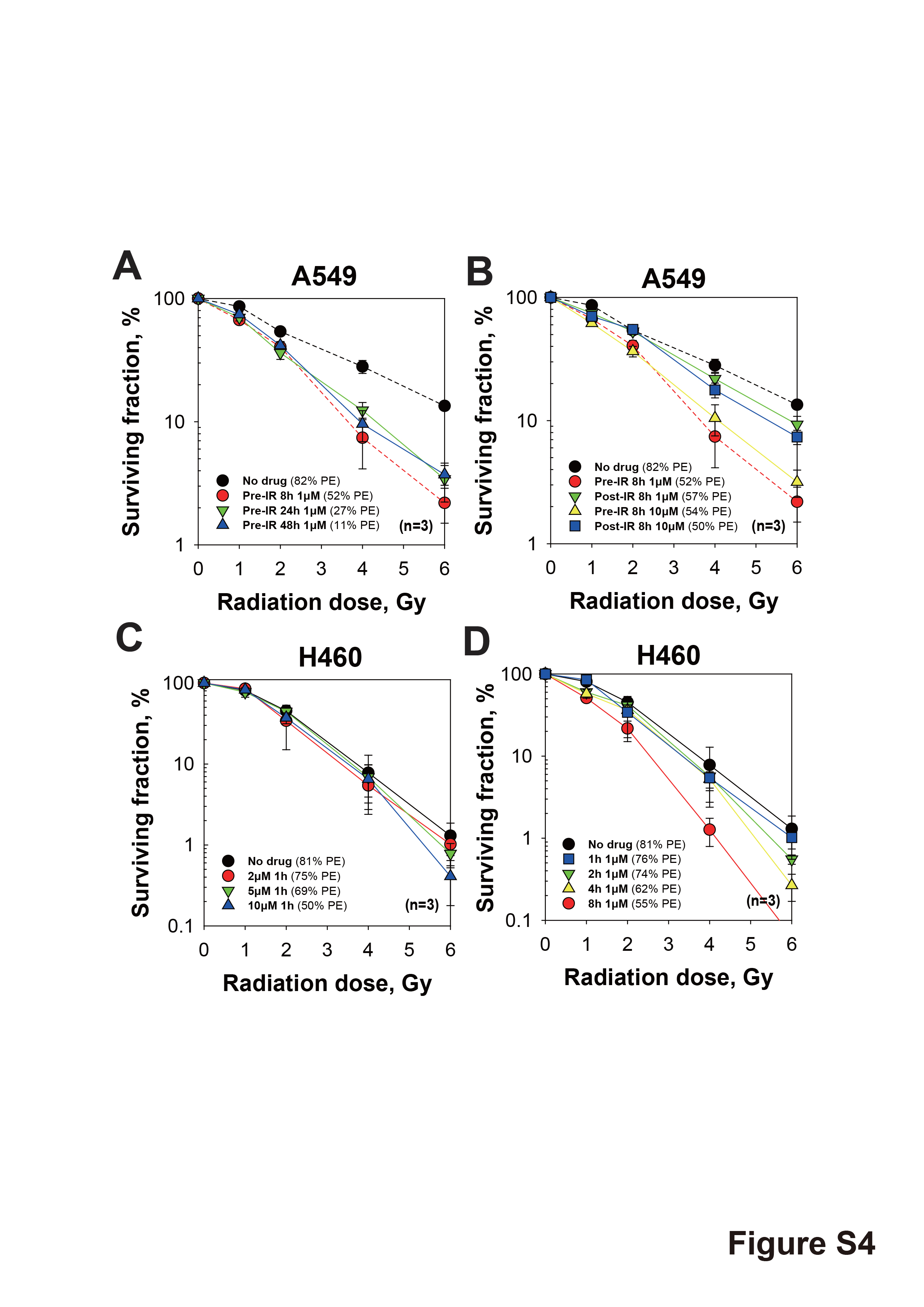

Supplement: Supplementary file 4 — Figure S4. Assessments of cisplatin‐mediated radiosensitization under different treatment conditions. [file MOL2-20-1814-s006.jpg]

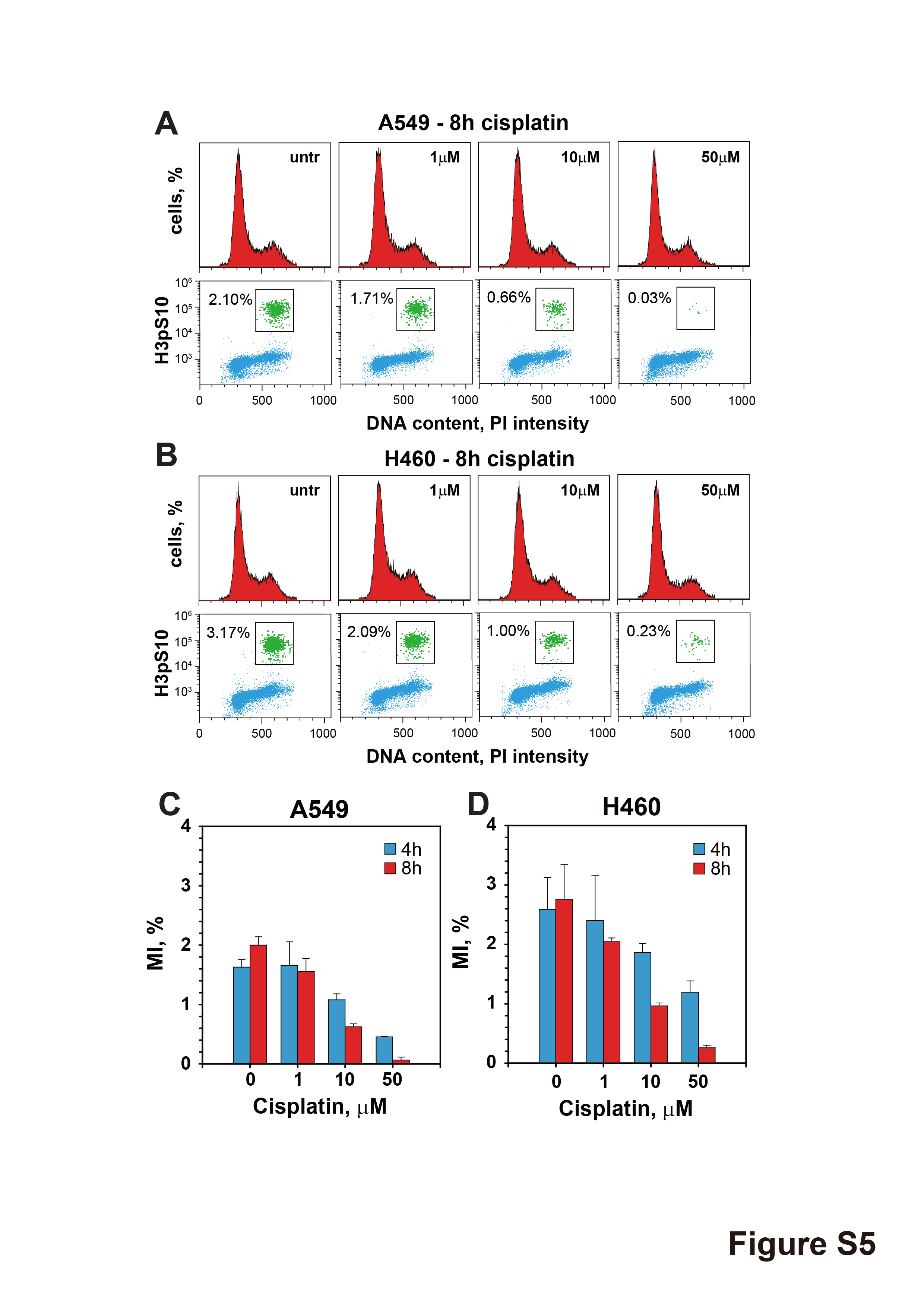

Supplement: Supplementary file 5 — Figure S5. Effects of cisplatin treatment of the distribution of cells throughout the cell cycle. [file MOL2-20-1814-s004.jpg]

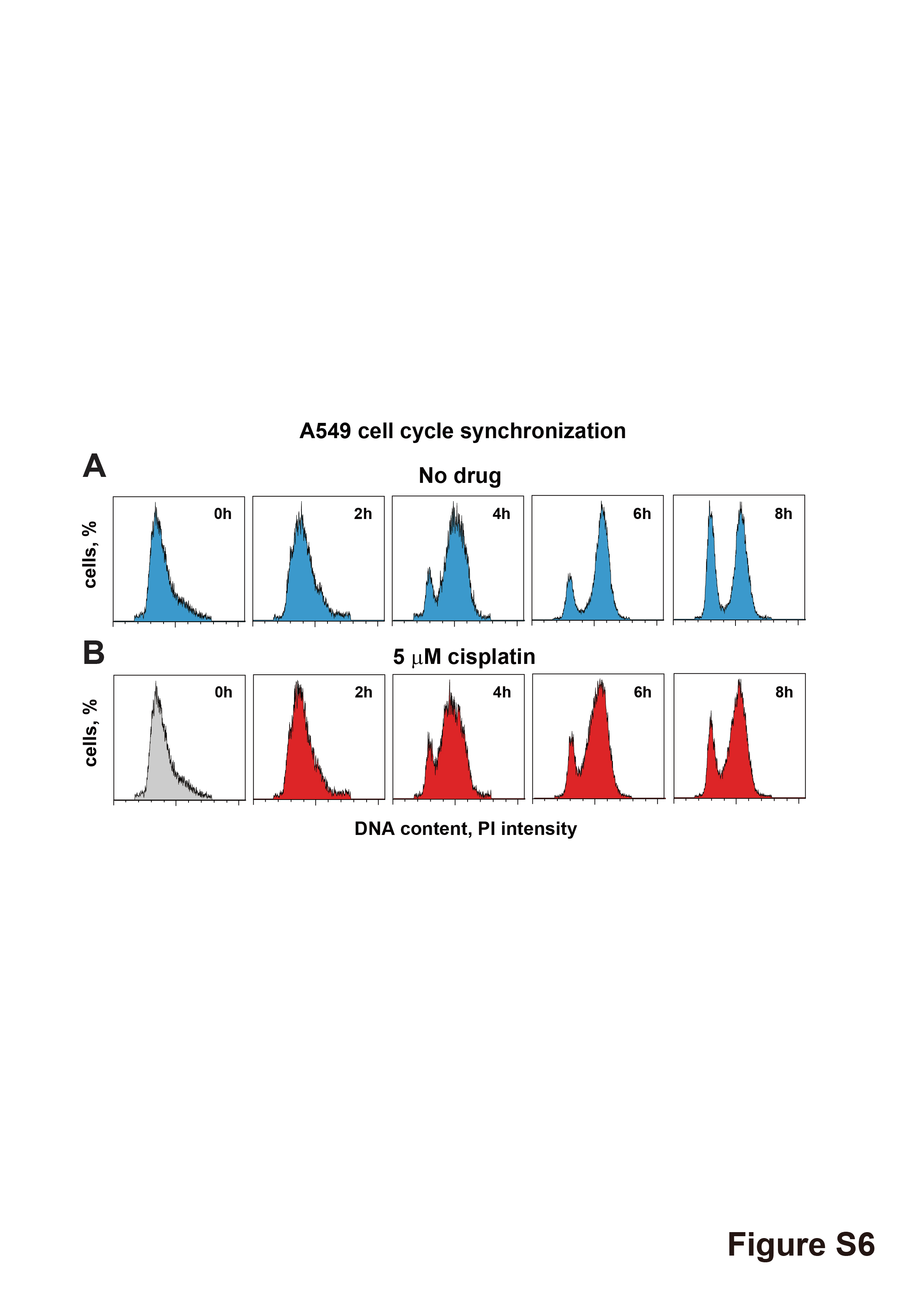

Supplement: Supplementary file 6 — Figure S6. Cell cycle progression after the release from thymidine block. [file MOL2-20-1814-s003.jpg]
