# Supplementary material for: Cell‐cycle‐specific lesion evolution rather than inhibition of double‐strand‐break repair underpins cisplatin radiosensitization
Source: Mol Oncol. 2026 Mar 19;20(7):1814–34. doi: 10.1002/1878-0261.70240 (PMC13352966; doi:10.1002/1878-0261.70240)
Supplement: Supplementary file 7 — Table S1. Drugs and antibodies. [file MOL2-20-1814-s005.docx]

**Supplementary Information**

**Cell-cycle-specific lesion evolution rather than inhibition of double-strand-break repair underpins cisplatin radiosensitization**

**Supplementary figure legends**

**Figure S1**: *Effects of Cisplatin on DSB induction and repair in H460 cells.* (A–C) Effects of 50 μM cisplatin on γH2AX foci induction and repair in H460 cells at the G_1_ (A), S (B), and G_2_ (C) phases of the cell cycle; (D) Dot plot of EdU labelling with relevant gates employed in the analysis; (E) Effects of 50 μM cisplatin on DSB repair kinetics in A549 cells exposed to 20 Gy and analyzed using PFGE; cisplatin was added 1 h before IR; (F) as in (E) for the effects of 50 μM cisplatin on H460 cells. Data of Figure A-C represent mean ± SEM calculated from one experiment, while data of Figure E and F represent mean ± SD calculated from three independent experiments in PFGE. Statistical analysis was performed between the no drug group and the 50 μM treatment group. The p-value was calculated using the two-tailed Student´s test. * p < 0.05, ** p < 0.01. n.s, nonsignificant.

**Figure S2**: *Cisplatin has no impact on DNA end resection*. (A) Representative images of RPA70 foci following 2 Gy IR and 50 µM cisplatin in A549 cells. Scale bar: 15 µm; (B–D) Effects of cisplatin on IR-induced RPA70 foci kinetics in A549 cells at the G_1_ (B), S (C), and G_2_ (D) phases of the cell cycle; Data represent mean ± SD calculated from two independent experiments.

**Figure S3**: *Cisplatin causes a delay on the formation of RAD51 foci*. (A–C) Effects of cisplatin on IR-induced RAD51 foci kinetics in A549 cells at the G_1_ (A), S (B), and G_2_ (C) phases of the cell cycle; (D-F) as in (A-C) for effects of 50 µM cisplatin in H460 cells at the G_1_ (D), S (E), and G_2_ (F) phases of the cell cycle. Data represent mean ± SEM calculated from one experiment.

**Figure S4**: Assessments of *cisplatin-mediated radiosensitization under different treatment conditions.* (A) Clonogenic survival in A549 cells demonstrating radiosensitization after continuous 1 μM cisplatin treatment for 8, 24, and 48 h before IR; (B) Radiosensitization of A549 cells following treatment with either continuous 1 μM cisplatin (8h) or pulse 10 μM cisplatin (1h + 7h recovery) administered before or after IR; (C) Radiosensitization assessed as in (A) for H460 cells treated with 2-10 μM cisplatin for 1h before IR; (D) Radiosensitization assessed as in (A) for H460 cells treated with 1 μM cisplatin for 1-8h before IR. Data represent mean ± SD calculated from three independent experiments.

**Figure S5**: *Effects of cisplatin treatment of the distribution of cells throughout the cell cycle.* (A, B) Representative histograms and dot plots obtained from FC; (C) Effects of cisplatin at different concentrations on the mitotic index (MI) in exponentially growing A549 cells; (D) as in (C) for H460 cells. Data represent mean ± SD calculated from two independent experiments.

**Figure S6**: *Cell cycle progression after the release from thymidine block*. (A, B) Representative histograms of cell cycle progression after release from thymidine block in A549 cells treated without (A) or with 5 μM cisplatin (B).

**Supplementary Table S1.** Drugs and antibodies

| **Drugs** | **Source** | **Cat. No** |  |
| --- | --- | --- | --- |
| Cisplatin | Teva® | - |  |
| Aphidicolin | MedChemExpress | HY-N6733 |  |
| **Antibody** | **Species** | **Dilution** | **Source** |
| γ-H2AX Ser139 | Mouse monoclonal | 1:400 | Gene Tex |
| 53BP1 | Mouse monoclonal | 1:400 | Abcam |
| H3pS10 | Rabit polyclonal | 1:2000 | Abcam |
| R-C18 | Rat | 0.2 µg/mL | Liedert, et al.2006 |
| RPA70B | Mouse monoclonal | 1:400 | Custom made and and  purified from mouse  hybridomas |
| Ki-67 | Rabbit polyclonal | 1:400 | Abcam ab15580 |
| AlexaFluor®488 | Goat anti-mouse | 1:400 | Life Tech. Corporation |
| AlexaFluor®488 | Goat anti-rabbit | 1:400 | Thermo Fisher Scientific |
| AlexaFluor®568 | Goat anti-mouse | 1:400 | Life Tech. Corporation |
| AlexaFluor®647 | Goat anti-mouse | 1:400 | Life Tech. Corporation |
